# The Role of Gender Information in Pronoun Resolution: Evidence from Chinese

**DOI:** 10.1371/journal.pone.0036156

**Published:** 2012-05-16

**Authors:** Lijing Qiu, Tamara Y. Swaab, Hsuan-Chih Chen, Suiping Wang

**Affiliations:** 1 Center for the Study of Applied Psychology, South China Normal University, Guangzhou, China; 2 Department of Psychology and Center for Mind and Brain, University of California Davis, Davis, California, United States of America; 3 Department of Psychology, Chinese University of Hong Kong, Hong Kong, China; University of Leicester, United Kingdom

## Abstract

Although previous studies have consistently demonstrated that gender information is used to resolve pronouns, the mechanisms underlying the use of gender information continue to be controversial. The present study used event-related potentials (ERPs) to investigate whether working memory modulates the effect of gender information on pronoun resolution. The critical pronoun agreed or disagreed with its antecedent in gender. Moreover, the distance between a pronoun and its antecedent was varied to assess the influence of working memory. Compared with the congruent pronouns, the incongruent pronouns elicited an N400 effect in the short distance condition and a P600 effect in the long distance condition. The results suggest that the effect of gender information on pronoun comprehension is modulated by working memory.

## Introduction

Two linguistic expressions are coreferential when they refer to the same semantic entity, and the process of establishing coreference between expressions is essential in extracting a coherent message-level representation of a text. The establishment of gender agreement is a critical component of coreferential processing. Previous studies have shown that both semantic (biological) and syntactic (linguistic) gender information have important roles in this process [1]–[8]. However, the cognitive mechanisms underlying the use of gender information remain subject to debate. Electrophysiological studies of the contribution of antecedent gender information to pronoun (anaphor) processing have produced contradictory results. Some studies suggest that it is driven by syntactic operations [9], [10], some indicate that it is resolved at the semantic level [11], and some others suggest that it involves both semantic and syntactic processing [12]. The present study used event-related potentials (ERPs) to further explore the cognitive mechanisms of the effect of gender information on pronoun resolution.

### ERP Effects of Gender Violations on Pronoun Comprehension Vary with Different Languages

Previous ERP studies of the influence of gender information on pronoun resolution have often used gender disagreement paradigms. In this paradigm, the gender of the pronoun is manipulated so that it agrees or disagrees with the gender or gender stereotype of the antecedent. The underlying cognitive mechanisms of pronoun comprehension are assessed by comparing the ERPs of the congruent and incongruent pronouns. Most previous ERP studies on pronoun understanding in English have reported an increased P600 to referentially failing pronouns, i.e., when the gender of the pronoun mismatched with the biological gender of the antecedent [9], [10]. For example, Osterhout and Mobley [9] found an enhanced P600 effect for pronouns that disagreed with the antecedents in gender when participants considered the sentences as unacceptable (e.g., “The successful woman congratulated himself…”; “The aunt heard that he …”). The P600 is observed when syntactic violations and syntactic ambiguity induce attempts to revise the syntactic structure [13]–[15]. Furthermore, increases in the amplitude of the P600 have been associated with costs that result from revisions needed to resolve a conflict between syntactic and semantic information, for example in syntactically correct and unambiguous sentences that violate thematic constraints [16]–[18], or more generally, to resolve a conflict by using information from language and non-language sources to achieve comprehension of the sentence [19], [20]. Because English pronouns do not have syntactic gender, Osterhout and Mobley [9] have suggested that the P600 found reflects a violation of the grammatical rule that pronouns must agree in gender with their antecedents.

Studies discussed thus far have explored ERP effects of biological gender violations on pronoun resolution in English. In a series of studies Münte and colleagues manipulated both biological and syntactic gender in German and Dutch [11], [12], [21]–[23]. In contrast to English, German and Dutch mark syntactic gender of nouns, and allow for manipulations of pronoun resolution only on the basis of syntactic gender agreement between the anaphor and its antecedent (i.e., the noun apple has a masculine syntactic gender and requires a masculine pronoun). The results of these studies were consistent for the syntactic gender manipulation, such that a P600 resulted when the anaphor violated the syntactic gender of the antecedent [11], [22], which is interpreted as that syntactic operations were involved during pronoun resolution. But when biological gender was manipulated in addition to syntactic gender, the pattern of results was less uniform: sometimes a P600 was observed [11], [21]–[23], sometimes an N400 [11], and sometimes a combination of both [12], [21]. The N400 is commonly considered to be sensitive to semantic aspects of the input, and its amplitude is reduced to words that can be easily integrated into the preceding context [24]–[27]. These results suggest that, at least under certain circumstances, conceptual/semantic integration is involved during pronoun interpretation. When personal pronouns violated the antecedents in only biological gender, only a P600 effect was revealed, which was interpreted as the involvement of syntactic analysis [21] or a morphosyntactic violation [28].

In the present study, we will manipulate gender agreement during pronoun resolution in Chinese, a language that differs in a number of characteristics from the languages used in the studies mentioned above. Chinese possesses very few linguistic gender markers and derivational inflections and has no inflections for tense or case. Very often the discourse will be gender-neutral (like saying police-person in English) and in spoken language only one third-person pronoun of the same pronunciation “TA” is used for “he”, “she” and “it”. In written language, pronouns are gender marked (although this is a relatively new addition to the language). Thus, in order to model cognitive mechanisms involved in establishing the gender agreement between a pronoun and its antecedent, these cross-linguistic differences should be taken into account. Even though pronoun resolution has been studied in Chinese [7], [29], [30], to our knowledge this is the first study of Chinese to explore the ERP effects of gender violation on pronoun processing.

### A Possible Role of Working Memory in the Effect of Gender Information on Pronoun Comprehension

The linking of a pronoun to its antecedent requires the availability of the antecedent in memory, and increasing the distance between the pronoun and its antecedent may increase demands on working memory (WM) operations. Clark and Sengul [31] provided some evidence for this idea. In their study, they manipulated the distance between the anaphor and the antecedent and found that the comprehension times increased with the distance. With a similar task, Streb et al. [32] reported a greater N400 for far distance compared with near distance pronouns.

Based on these findings, Hammer and colleagues [11] hypothesized that a larger distance between a pronoun and its antecedent would increase WM demands during pronoun comprehension, because the information about the antecedent decays and will be more difficult to be re-activated. Attempting to examine whether the effect of gender information on pronoun processing could be modulated by WM, in a clever design they manipulated the distance between a pronoun and its antecedent and included purely syntactic gender manipulations (i.e., the thing condition) and manipulations of both biological and syntactic gender (i.e., the person condition). Consistent with their previous findings, they again showed a P600 effect for the syntactic gender disagreement in the short distance (SD) condition, but they did not find any violation effect in the long distance (LD) condition which they interpreted as indicating that syntactic gender information decays over time. In contrast to most previous findings in English, but consistent with some of their own previous findings (as discussed in the previous section), they found a very early negative ERP effect of gender incongruency in the SD-person condition. They interpreted this as an early-onset N400 effect and suggested that the establishment of coreference between a person pronoun and its antecedent is semantically driven in German. The results for gender incongruency in the LD-person condition varied as a function of the way in which the distance was manipulated. In one condition, the material intervening between the pronoun and its antecedent contained a subject-gap (e.g., “The chief attacks soon and [gap] is martial, because he/she wants to win.”), and the authors suggested that the antecedent information was likely re-activated when this gap was encountered, effectively reducing the distance; in this condition they found a P600 effect. In the other LD condition, there was no subject gap, and here they found an N400 effect.

In general, the authors suggested that their pattern of results indicates that gender agreement in the thing condition relied on syntactic operations, but when the antecedent was a person, and the violation was also one of biological gender, gender congruency relied on semantic operations. This is interesting because it indicates that a language that has clear case and gender marking will separate syntactic gender violations (P600) from biological gender violations (N400), perhaps because the animacy clue is more salient than the syntactic cue. However, their interpretation does not appear consistent with the finding of a P600 in the LD-person condition containing a subject gap. If this subject gap indeed re-activated the antecedent, as the authors suggested, then the distance would effectively be similar to that in the SD condition, and this would predict an N400 effect in this condition as well. Thus the finding of a P600 in the LD gap condition is somewhat puzzling. Furthermore, the antecedent in this study was always located in the clause that preceded the clause with the pronoun, and the distance was manipulated by adding a proposition or adverbial modifier to this clause. This may be non-optimal because previous findings suggest that distance effects occur when the pronoun is separated from the antecedent by an intervening clause [31].

In sum, previous ERP studies are inconclusive with respect to the nature of the processes that underlie the biological gender agreement between the antecedent and the anaphor. It is possible that the nature of the Chinese language will shed further light on this. It would also be interesting to see if the findings in the Chinese language mirror the German studies more, because Chinese lacks syntactic gender marking, even more so than English does. Accordingly, in the present study we manipulated both (biological) gender agreement and distance between a pronoun and its antecedent to further assess the cognitive mechanisms of the influence of (biological) gender information on pronoun resolution.

### The Present Study

Participants read sentences in Chinese about a person (the antecedent) that contained a pronoun that was congruent or incongruent with respect to the gender stereotype associated with the antecedent name. Noted that although most personal names are unique to males or females in English, names in Chinese are not exclusively male or female but tend to be associated with a particular gender to varying degrees. The pronoun followed the antecedent in an immediately adjacent clause (short distance  =  SD condition) or after one intervening clause (long distance  =  LD condition). We examined whether this distance manipulation affected the nature of the processes involved in the establishment of gender agreement between the pronoun and its antecedent as reflected by the N400 and the P600. Consistent with previous findings [9], [10], [28], we could observe a P600 effect for gender disagreement in the SD condition, suggesting the involvement of syntactic processes, which could be reduced or delayed in the LD condition because the gender information of the antecedent name is less available. However, because Chinese pronouns do not contain syntactic gender information or case marking, the comprehension of Chinese pronouns may be based on semantic operations under all distance conditions. This leads to another possible outcome that an N400 would be found for gender mismatch in both the SD and LD conditions, but that the N400 effect in the LD condition would differ in terms of amplitude or latency because of reduced availability of the antecedent.

## Materials and Methods

### Ethics Statement

This study was approved by the Psychology Research Ethics Committee of South China Normal University. The participants provided written informed consent prior to the experiment.

### Participants

The participants were 18 college students (4 males; ages 19–24 years, mean 20.5 years) from South China Normal University who were paid for their participation. All were right-handed native Chinese speakers with normal or corrected-to-normal vision. None had any neurological impairment.

### Materials and Design

In a pretest, participants rated the extent to which 558 Chinese names were associated with a particular gender using a 5-point rating scale (1 for extremely female and 5 for extremely male). Based on this pretest, 160 names were selected for sentence construction. Half of the names were stereotypically female (average rating <1.33, standard deviation = 0.19), and half of the names were stereotypically male (average rating >4.48, standard deviation = 0.20).

A total of 160 stimulus pairs were constructed, and each pair included one of the 160 names. Each trial contained an initial sentence with an antecedent noun (i.e., a name) serving as the subject, followed by a target sentence of which the subject was a coreferential pronoun (see Table 1). The gender of the pronoun was manipulated so that the pronoun agreed or disagreed with the gender stereotype of the antecedent. Moreover, the distance between the pronoun and its antecedent was either short or long. In the SD condition, the initial sentence consisted of two clauses with the antecedent in the second clause, and the target sentence with the pronoun consisted of one clause. In the LD condition, the initial sentence contained one clause, and the target sentence contained two clauses with the critical pronoun in the second clause. Because both the antecedent and the pronoun were in the subject position, this distance manipulation did not generate any difference in the syntactic complexity or syntactic prominence of the antecedent across conditions. The experimental manipulations resulted in a 2×2 design and the two factors were congruency (congruent/incongruent) and distance (SD/LD).

**Table 1:** Example materials.

Condition	Sentence
SD-C	在新一轮优秀个人评比活动中, **潘振**获得了好评.**他**以绝对优势当选劳模.
	In the new round of competition for the outstanding individual, **Panzhen** (male name) earned acclaim. **He** won the Model Worker award with an absolute advantage.
SD-I	在新一轮优秀个人评比活动中,**潘振**获得了好评.**她**以绝对优势当选劳模.
	In the new round of competition for the outstanding individual, **Panzhen** (male name) earned acclaim. **She** won the Model Worker award with an absolute advantage.
LD-C	**潘振**获得了好评.在新一轮优秀个人评比活动中, **他**以绝对优势当选劳模.
	**Panzhen** (male name) earned acclaim. In the new round of competition for the outstanding individual, **he** won the Model Worker award with an absolute advantage.
LD-I	**潘振**获得了好评.在新一轮优秀个人评比活动中, **她**以绝对优势当选劳模.
	**Panzhen** (male name) earned acclaim. In the new round of competition for the outstanding individual, **she** won the Model Worker award with an absolute advantage.

The example sentences are in Chinese with English translations. The critical personal names and pronouns are in bold. The “bold” here is for demonstration only but not in the experimental presentation. SD, short distance; LD, long distance; C, congruent; I, incongruent.

Four lists were constructed, each consisting of 40 items in each of the four conditions. Only one condition of each item was presented in a list. Half of the target pronouns in each list matched the gender stereotype of the antecedent names, and the other half did not. Furthermore, half of the antecedent names in all four conditions were stereotypically female, and half were stereotypically male. Each list included 80 filler items with a similar structure to the test items, intermixed with the test items in a fixed pseudorandom order. The filler stimuli did not incorporate any type of anomaly. To keep participants attentive and ensure that they comprehended the sentences, they answered yes-no comprehension questions for 58 filler stimuli. For the purpose of avoiding undue emphasis on the experimental manipulation, none of these questions probed the pronominal aspects of the sentences. Half of the questions required a “yes” response, and half required a “no” response.

### Experimental Procedure

Each participant was tested in a dimly lit, electrically shielded, and sound-attenuating room while sitting in a comfortable chair. The sentences were presented on a computer screen that was located approximately 100 cm in front of the participants. The participants were instructed to read the sentences silently and carefully and to answer a yes/no question when prompted. They were asked to remain still and to refrain from blinking and moving their eyes while reading to avoid artifacts in the ERPs. However, they were allowed to blink between sentences and when responding to questions. Each trial started with a fixation cross presented at the center of the screen for 300 ms. The initial sentence and the target sentence were then presented word by word in white characters against a black background in Kai-Ti_GB2312, 19-point font. Before the experiment we had asked four college students majoring Chinese from South China Normal University to do the word segmentation. When there are inconsistencies about some segments, we presented those segments in the smallest unit. The words for both the initial and target sentences were presented at a rate of 400 ms with an inter-stimulus interval of 250 ms. The comma for the initial sentence or the target sentence, and the period indicating the end of the initial sentence were presented separately for 400 ms followed by a blank screen for 250 ms after the comma, and 500 ms after the period. There was a six-sentence practice block to familiarize the participants with the procedure.

### EEG Recording and Data Analysis

The EEG was recorded from 38 electrodes fitted in an elastic cap (Quik-Cap) at the following locations: FPz, Fz, FCz, Cz, CPz, Pz, POz, Oz, FP1/2, AF3/4, AF7/8, F3/4, F7/8, FC3/4, FT7/8, C3/4, T7/8, CP3/4, TP7/8, P3/4, P7/8, PO5/6, and PO7/8. The EEG was referenced to the left mastoid online, and the right mastoid was recorded as an active channel for off-line re-referencing to the mean of the right and left mastoids. Vertical eye movements and blinks were registered by electrodes at the sub- and supraorbital edges of the left eye. The AFz electrode served as ground. Electrode impedances were maintained below 5 kΩ. The EEG was amplified with BrainAmps DC amplifiers (BrainProducts), sampled at 500 Hz and filtered with a 0.016 to 30 Hz bandpass. The ERP responses to the pronouns were measured in epochs of 1200 ms with a 200 ms prestimulus baseline. Each epoch was automatically screened for artifacts, such as eye movements. On average, 8% of the trials were discarded because of artifacts (amplitudes larger than ±80 µV) with no differences observed across the conditions.

Because our main interest was to find out whether the congruency effect differs with different distances between a pronoun and its antecedent, ANOVAs were performed to separately evaluate the effect of gender congruency for the SD and LD conditions. These analyses were performed on the mean amplitudes in the 350–500 ms and 500–750 ms time windows after pronoun onset, with two factors of congruency (congruent/incongruent) and electrode site (34). Electrode sites FP1, FP2, FPz and Oz were not included because of excess drift or noise. Greenhouse-Geisser corrected p-values are reported for comparisons with more than one degree of freedom in the numerator [33].

## Results

### Comprehension Task

The mean accuracy for the comprehension task was 95% (range 91–100%) and this high level of performance indicated that participants comprehended well the sentences.

### ERP Data

**Figure 1:**
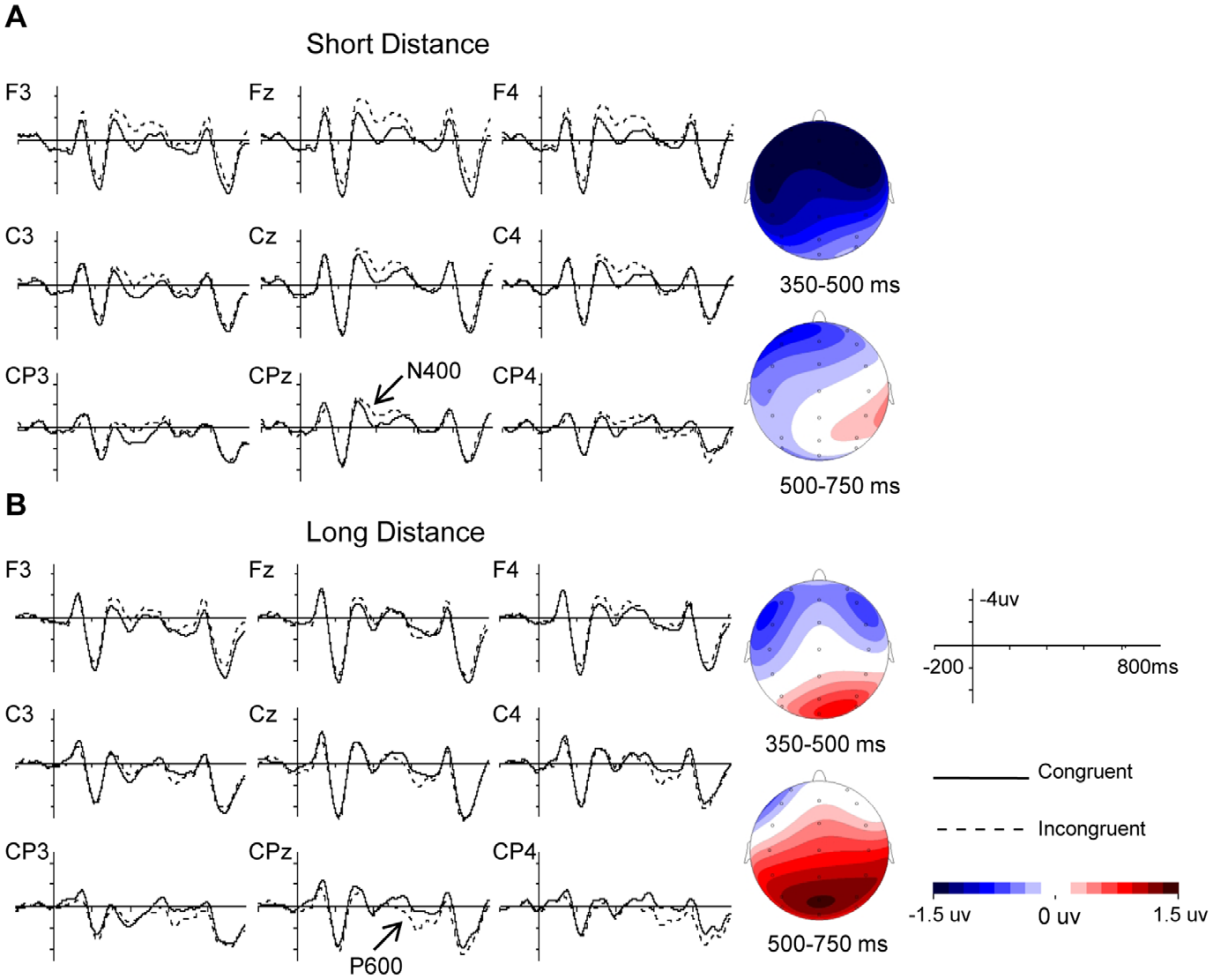
Grand average ERPs to pronouns for the congruency effect and corresponding scalp distributions. Panel A shows the comparison of congruent and incongruent pronouns in the short distance condition and the voltage maps display the mean difference effect (incongruent minus congruent pronouns) in the 350–500 ms and the 500–750 ms time windows. Panel B shows the same information for the long distance condition.

Grand average ERPs time locked to the onset of the pronouns and the corresponding scalp distributions for the congruency effect are shown in Figure 1. All conditions elicited an N1-P2 complex, which is typical to visually presented stimuli. Furthermore, a larger negative shift was seen for incongruent than congruent pronouns in the SD condition. In contrast, incongruent pronouns in the LD condition elicited a larger positive shift.

#### 350–500 ms

Table 2 presents the results of the ANOVAs. A separate ANOVA of congruency (congruent vs. incongruent sentences) in the SD condition showed a main effect of congruency (F(1,17) = 9.58, p<0.01), but did not reveal an interaction between congruency and electrode (F<2); thus, the results indicate that incongruent pronouns were more negative than congruent pronouns and that this effect had a widespread distribution, even though the topographic map in Figure 1 appears to indicate that the effect is maximal at anterior electrode sites. In previous studies of pronoun resolution, a frontally distributed negative shift has been observed to ambiguous pronouns with more than one antecedent. This effect has been labeled the Nref, and is presumed to index WM demands associated with maintaining referent information prospectively [28], [34]. As discussed in our methods section, our rating study of the antecedent names used in this experiment had shown that half were stereotypically female and half were stereotypically male, and this makes it unlikely that the antecedent was perceived as ambiguous. In addition, previous studies of gender disagreement have either shown P600 or N400 effects. Thus, we conclude that the effect of congruency observed here is most likely an N400 effect.

**Table 2:** Mean ERP amplitude ANOVAs for pronoun position.

Source	*df*	*F*-value (350–500 ms)	*F*-value (500–750 ms)
SD: Congruency	1,17	9.58**	0.7
SD: Congruency × Electrode	33,561	1.81	1.45
LD: Congruency	1,17	0.14	2.05
LD: Congruency × Electrode	33,561	2.57*	3.38*

SD, short distance; LD, long distance. Congruency (congruent vs. incongruent);

* p<0.05;

** p<0.01.

There was no main effect of congruency in the LD condition (F<1), but there was a significant interaction of congruency and electrode (F(33,561) = 2.57, p<0.05). However, further pair-wise t-tests for the congruency effect per electrode site indicated that the congruency effect was only marginally significant at electrode sites PO6 (t(17) = 2.01, p = 0.06) and PO8 (t(17) = 1.78, p = 0.09). Inspection of the ERPs in Figure 1 suggested some gender congruency effects at some electrode sites in the LD condition. In order to test whether there were any significant differences, we performed t-tests per electrode site across several different time windows. These time windows were 300−400 ms, 300–450 ms, 300–500 ms, 350–450 ms and 350–500 ms. The results of these analyses showed no significant differences between the congruent and incongruent conditions in all of the time windows, except at two electrodes of F3 and FC3 in the time window of 300–400 ms. Therefore, we argue that there is not enough evidence to reveal a difference in the N400 between the congruent and incongruent conditions for LD sentences.

#### 500–750 ms

The comparison of congruency for the SD condition did not reveal a main effect of congruency (F<1) or a congruency by electrode interaction (F(33,561) = 1.45, p>0.1). Moreover, further t-tests did not show any congruency effect at any electrode site. Thus the results revealed no difference in the P600 between the congruent and incongruent pronouns in the SD condition. For the LD condition, there was an interaction between congruency and electrode (F(33,561) = 3.38, p<0.05), which reflected a greater positivity for incongruent relative to congruent pronouns at posterior sites.

## Discussion

The present study investigated the cognitive mechanisms of personal pronoun resolution in Chinese, a language that does not syntactically mark gender, and gender agreement is established on the basis of biological gender. The target pronoun was congruent or incongruent with the gender stereotype of its antecedent. Moreover, the clausal distance between a pronoun and its antecedent was changed to manipulate WM. Comparing the ERPs to gender incongruent with congruent pronouns for different distance conditions, we explored whether the establishment of gender agreement between a pronoun and its antecedent can be modulated by the demands on WM. Contrary to our predictions and the findings by Hammer et al. [11], the current results showed an N400 effect for gender incongruency in the SD condition, but a P600 in the LD condition. More specifically, incongruent pronouns elicited an increased N400 effect relative to congruent pronouns in the SD condition. This negative shift had a widespread distribution and was similar to the effects found by Lamers et al. [12] and Hammer et al. (in their SD-person condition) [11]. Consistent with previous findings of an N400 effect for gender disagreement on pronouns [11], [12], [21], the N400 found in the present study suggests the involvement of semantic analysis in the SD pronoun processing in Chinese.

In contrast, a P600 effect was found for gender disagreement in the LD condition. This finding is consistent with previous findings of a P600 for biological gender disagreement [9], [10], [28]. It has been suggested that this P600 effect reflects a violation of a grammatical rule that pronouns must agree in gender with their antecedents [9], or as being the result of a morphosyntactic violation [28]. It is important to note, however, that most experiments of Osterhout and colleagues [9], [10] violated (biological) gender agreement of reflexive pronouns which may constitute purely syntactic violations – the antecedent of a reflexive pronoun has to occur within the same sentence [35]. Nieuwland and Van Berkum [28] found an enhanced P600 when readers encountered a pronoun that was inconsistent with the bias of the verb (e.g., “Linda apologized to David because he…”) relative to bias-consistent pronouns, even though a suitable antecedent was present in the preceding sentence context; hence the P600 may have reflected the cost of conflict between the outcome of the semantic analyses (i.e, there is not a suitable person to integrate the pronoun with) and the need to revise the syntactic structure to achieve referential agreement when the initial preferred interpretation of the sentence failed. In this same study they also included a gender violation manipulation, as in “Anna shot at Linda as he jumped over the fence”, and also found an enhanced P600. They suggest that this finding shows that syntax takes the blame when there is no antecedent that matches the pronoun in gender. But since these sentences were presented in the same experiment as the sentences that contained the verb bias manipulation, it is possible that participants also attempted to revise the syntactic structure after the failure of the semantic analyses.

The P600 effect for gender disagreement in the LD condition of the present study can not easily be explained in terms of syntactic processing difficulties, since there is no syntactic gender in Chinese. A personal pronoun must agree with the semantic (biological) gender of its antecedent, and the establishment of gender agreement between a personal pronoun and its antecedent is semantic in nature in Chinese. Furthermore, it is difficult to explain why the effect of gender agreement would affect semantic processing in the SD condition, but would become grammaticalized at a greater distance, also in light of the findings of Hammer and colleagues [11] that the P600 effect of purely syntactic gender violation is not found with a greater distance between the pronoun and its antecedent.

However, many recent ERP studies have observed P600 effects when a conflict arises between semantic and syntactic analyses of the language input. On the basis of these findings, Kuperberg proposed a dynamic framework [36], suggesting that two distinct but interactive processing streams operate during online language comprehension: a semantic memory-based mechanism computing the semantic relationships between words, and a syntactic-based combinatorial mechanism combining words into propositions based on syntactic constraints. In this framework, the N400 reflects the activities of the semantic memory-based stream, and the P600 is evoked when there are conflicts between the outputs of these two streams. This framework does not offer a specific explanation under which circumstances semantic violations elicit N400 or P600 effects. We can therefore only tentatively explain why gender incongruency engendered an N400 in the SD condition but a P600 in the LD condition.

It has been suggested that the representation of the antecedent decays over time and becomes less available with increased distance [31], [37]. If this is the case, then the antecedent in our study would be more available in the SD than LD sentences. Because of the higher availability of the antecedent, the violation of biological gender would be much more salient in the SD than in the LD condition, leading to the N400 effect of gender incongruency in the SD but not in the LD condition. The point that the gender violation is larger in the SD than in the LD condition can to some extent be supported by the fact that a larger negative shift was elicited with the SD incongruent condition than with the LD incongruent condition in the current study. The semantic violation would be much less salient in the LD condition, since the activation of the antecedent has decayed, and this might allow the semantic memory-based stream to proceed normally, leading to a temporary alternative interpretation that the pronoun is not related to the mentioned entity but to an unmentioned entity. However, the syntactically determined combinatorial analysis interprets the pronoun to refer to the only mentioned entity and identifies the gender disagreement. Thus, a conflict occurs between the representations of these two processing streams and a P600 is elicited.

Noted that technically the pronoun is free and can refer to an unmentioned entity. However, for sentences used in the current study, in general the pronoun is thought to be interpreted to refer to the entity presented rather than an unmentioned entity and thus the gender incongruency appears. The reasons are as follows. First, the interpretation that the pronoun refers to the entity mentioned is preferable according to findings from other reading studies [38]. Second, the pronoun in isolated sentences is not absolutely free and can only be related to the entity mentioned because there are no alternative entities [21]. Moreover, the researcher argues that the emergence of the N400 or P600 for gender disagreement indicates that finally the reader recognized the referent of the pronoun is the only entity mentioned and there is a gender disagreement between the pronoun and its antecedent [9], [21], [28].

Kolk and colleagues [39] have proposed a somewhat different interpretation of the P600 effect. According to their monitoring hypothesis, the P600 effect might reflect a monitoring process that triggers a reanalysis of the input to check for possible processing errors when there is a conflict between representations that result from a plausibility heuristic computing the semantic associations between words and the syntactic parser. In this framework, the absence of an N400 effect in the LD condition could be explained as a function of heuristic processes leading to a temporary plausible interpretation that the referent of the pronoun might be out of the context. The P600 in the LD condition would be elicited because the syntactic parser recognizes the coreferential dependency between the pronoun and the antecedent person in the actual text, resulting in the gender incongruency effect, which conflicts with the outcome of the heuristic process, and this would trigger re-analyses.

In summary, our results demonstrate that the influence of biological gender information on Chinese pronoun resolution can be modulated by WM. We found an N400 effect for gender disagreement in the SD condition but a P600 effect in the LD condition. Based on the current results, we assume that Chinese personal pronoun resolution is primarily based on semantic operations and that a greater distance causes increased decay of the semantic representation of the antecedent in WM. Because the functional interpretation of the P600 is still debated and there is lack of ERP evidence of Chinese pronoun processing, to date a precise and specific explanation about the functional significance of the P600 in the LD condition and how WM affects the use of gender information can not be provided. More studies on the cognitive mechanisms of the role of gender information in pronoun resolution should be conducted to clarify this question.

Overall, the present study provides (a) evidence for the view that the effect of gender information on pronoun resolution can be modulated by WM and (b) new information about the electrophysiological signature of the role of gender information in pronoun resolution in reading Chinese. Thus, the present findings contribute to our understanding of both reading comprehension in Chinese and the processing of pronouns.
